# Inequality of health stock and the relation to national wealth

**DOI:** 10.1186/s12939-019-1096-x

**Published:** 2019-12-02

**Authors:** Isma Addi Jumbri, Shinya Ikeda, Masayuki Jimichi, Chika Saka, Shunsuke Managi

**Affiliations:** 1 Graduate School of Environmental Studies, Tohoku University, Sendai, Japan; 2 Faculty of Technology Management and Technopreneurship, University Teknikal, Melaka, Malaysia; 3 College of Agriculture, Regional and Environmental Science, Ibaraki University, Tsuchiura, Japan; 4 School of Business Administration, Kwansei Gakuin University, Nishinomiya, Japan; 5 Department of Urban and Environmental Engineering, Kyushu University, Fukuoka, Japan; 6 Urban Institute, Kyushu University, Fukuoka, Japan

**Keywords:** Health inequality, Gini coefficient, Inclusive wealth, Sustainability and national wealth

## Abstract

**Background:**

The decline in global and between-country health inequality is a major challenge to overcome. However, few studies have systematically investigated the relationship between inequality of health stock and national wealth. From an economic perspective, health can be viewed as a durable capital stock that produces an output of healthy time. Therefore, in this paper, we focused on health capital to investigate the relationship between inequalities of national health and national wealth.

**Methods:**

Based on health stock data from 1990 to 2015 for 140 countries, we estimated Gini coefficients of health stock to investigate associations with a well-known economic flow indicator, Gross Domestic Product (GDP), stock-based national wealth indicator, Inclusive Wealth Index (IWI), and firm-level net income.

**Results:**

The estimated Gini coefficient of global health stock shows that health stock has experienced a global decline. The Gini coefficient for low-income countries (LICs) showed the fastest decline in health stock, dropping from 0.69 to 0.66 in 25 years. Next, rapid population growth and the rise in the youth share of the working-age population in LICs were most likely contributing factors to the decline in inequality. Most countries that experienced positive health stock growth also indicated a strong positive relationship with GDP and IWI. However, some countries showed a negative relationship with natural capital, which is a part of IWI. In addition, firm-level net income showed no obvious associations with health stock, GDP and IWI.

**Conclusions:**

We argue that a negative relationship between health stock and natural capital is a sign of unstable development because sustainable development involves maintaining not only GDP but also IWI, as it is a collective set of assets or wealth comprising human, produced and natural capital. Moreover, in our analysis of firm-level income data, we also discuss that income will be influenced by other factors, such as innovations, human resources, organization culture and strategy. Therefore, the paper concludes that health stock is a vital component in measuring health inequality and health-related Sustainable Development Goals (SDGs). Thus, IWI is more comprehensive in measuring national wealth and can complement GDP in measuring progress toward sustainable development.

## Background

Health stock is a concept from the health economics literature pioneered by Michael Grossman [1, 2]. The Grossman model is widely used to explain how health is produced and viewed as a durable capital stock that produces an output of healthy time; each individual is regarded as both a producer and a consumer of health. Health is treated as a stock which degrades over time in the absence of “investment” in health [3, 4]. This model also states that the individual inherits an initial amount of stock of health capital that can depreciate with age or be increased through investment [5–7]. In Grossman’s framework, an individual is assumed to inherit an initial stock of health. However, the accumulation of this stock depends on the entire history of past resources, behavior and consumption. Therefore, health stock may grow, decline, or remain constant over time. For instance, health stock will decline with age and can be increased with investment in one’s health, such as by purchasing preventive and curative medical care [6].

Inequality of health stock in a nation would signal an unsustainable economy. Several studies have highlighted that accounting for health capital stock is important to achieving sustainability because health stock is a vital resource to sustain human well-being, which is usually represented by national wealth indicators. For instance, Arrow et al. [8, 9] and UNU-IHDP and UNEP [10, 11] highlighted the importance of health stock as a vital component of global sustainable development that should be consistently included as a stock base in measuring national wealth and sustainability. Furthermore, Ikeda et al. [12] in Japan found that health capital has significant impacts on regional sustainability. Furthermore, the increment of health stock indicates a positive signal for sustainability [13]. From the perspective of policy debates, improving health is both a benefit of and a prerequisite for achieving Sustainable Development Goals (SDGs) [14, 15]. According to the trend, inequality in health stock and its relation to national wealth must be analyzed, particularly in contributing to SDG 3 and 10, which focus on healthy lives and well-being, and on inequality. In addition, we could reduce the efforts to measure economic indexes related to national wealth for policymakers if health stock was a vital sign of an unsustainable economy.

In this study, we focused on the Inclusive Wealth Index (IWI) as the newest indicator of national wealth, which includes the value of health stock. IWI undoubtedly stands out as one of the most promising endeavors among recent high-profile new indicators of national wealth that leads to sustainability [16]. In the framework of IWI, at its minimum, sustainability requires the simultaneous preservation not only of human capital, comprising education and health but also of produced capital related to manufactured assets such as roads and machines, and natural capital involving forests and fossil fuels [17]. Moreover, health as a capital stock can be measured using a method initiated by Arrow et al. [13]; that is, the amount of health stock can be measured by calculating the total discounted years of life expectancy in a national population.

The IWI framework also highlighted several prominent issues. First, the environmental and natural ecosystem in wealth accounting is important [18]. Hence, a country must maintain nondeclining welfare over time by including natural capital in measuring capital stock or wealth to maintain a sustainable path. The second important issue is global responsibility and the effect of the individual on achieving sustainable development worldwide [19]. In this study, IWI is defined as the sum-value of a country’s capital asset stock (including produced, human and natural capital). In this regard, the associated economic theory states that development is sustainable if this sum-value does not decline through time [20, 21] and that each capital is evaluated based on its shadow price [10–12]; this notion is based on studies using IWI as a measurement to assess sustainability development and wealth in global, country and regional settings. Thus, the use of IWI to measure the relationship between health stock and socioeconomic status (SES) along with GDP provides a more complete picture of inequality in contemporary societies across the world [11].

To date, several studies have compared health inequality among different countries, taking into account aspects such as SES or national wealth. For instance, a study by Palafox, McKee and Yusuf [22] measured the patterns of wealth-related health inequalities in term of awareness, treatment and control of hypertension in 21 countries. In addition, a study by Houweling et al. [23] measured health inequality in maternal and child health in 43 countries and investigates the relation of SES to inequalities. However, limited studies are available on health stock and its relationship to national wealth (both GDP and IWI) and firm-level net income, information that appears difficult to obtain but could be an alternative indicator of national wealth. Therefore, understanding the relationship between health, national wealth and firm-level net income could produce important policy implications for sustainable development, related to SDG 3 and SDG 10. Looking beyond GDP and adopting IWI by tracking the evolution of the stocks of produced capital, natural capital, and human capital over time will help guide policymakers in their decisions relevant to sustainable development. Hence, this research contributes to the formation of criteria for health-related sustainability.

In this study, as the proxy of firm-level sustainability and to identify the effect of health stock on it, firm-level data were used comprising firm-level net incomes of 136 countries that had survived for over 100 years and achieved sustainability. The use of firm-level data allowed us to overcome the limitations of aggregate data and avoid differences or changes in macroeconomic factors [24, 25].

The remainder of the paper is organized as follows. The next section describes the methodology and dataset used to model the health stock and the measurement of health inequality in health stock using the Gini coefficient. The fourth presents the results of the empirical analysis. The last section provides summaries and discusses the implications of this study in terms of how the SDGs can be achieved.

## Methods

Before measuring inequalities in health, we explain why we use the method proposed by Arrow et al. [8] to calculate the amount of health stock for 140 countries from 1990 to 2015. Unlike commonly used indicators, e.g., life expectancy at birth or Body Mass Index, this indicator is supported by rigorous economic theory to represent the capital form of human health, that is, monetarized indicators. Moreover, the monetarizing process allows for a comparison with other type of capital, such as manufactured capital, natural capital, even GDP. Using this method, the amount of health stock can be calculated using total discounted years of life expectancy for each age group in a country’s population. Note that to monetarize health stock we multiply the amount of health stock and the value, or shadow price, which couldn’t be observed in the market. Therefore, we need to estimate the shadow price and use the value of an additional year of life, the value of statistical life (VSL), as a basement of the shadow price, though the shadow price is assumed to be constant for each country during our estimated period.^1^ We briefly explain this application because we used the health stock data calculated in and more detailed descriptions are available in Jumbri et al. [13].

We now explain how to calculate the amount of health stock. Let π(*a*) be the proportion of people of age *a*, conditional probability density, *f* (*T*), resulting from computing the probability density that someone born will die at age *T*, and the corresponding cumulative distribution at age *a*, *F*(*a*). The conditional probability density of death at age *T*, given survival to *a*, *f*(*T*| *T* ≥ *a*) can be represented as follows:

$$ f\left(T|T\ge a\right)=\frac{f(t)}{1-F(a)} $$

We assume that *δ* is a constant discount rate for future survival years and the value of additional survival years is not correlated with age. We also assume that all individuals would die before the age of 100 due to data availability.^2^ The amount of health capital stock per capita at age *a*, *H*(*a*), can then be calculated as follows:

$$ H(a)={\sum}_{a=0}^{100}\pi (a)\left\{\sum \limits_{T=a}^{100}f\left(T|T\ge a\right)\left({\sum}_{t=0}^{T-a}{\left(1-\delta \right)}^t\right)\right\} $$

Subsequently, the total amount of health stock can be calculated by summing it up as $\sum \limits_{a=0}^{100}H(a)$ in the total population of a country. The data on the probability of death at age *t*, *f*(*t*) by five-year age intervals, are obtained from the life tables, which include the number of people dying between ages *x* and *x* + *n* in each year from 2000 to 2015. The data for the years 1990, 1995, 2000, 2005, 2010 and 2015 were available from the database of the country-level life tables of WHO (http://apps.who.int/gho/data/node.imr. LIFE_0000000032?lang = en). We impute missing data of the probability of death at each age group by linear interpolation in 1990–1994, 1996–1999, 2001–2004, 2006–2009, and 2011–2015. The rest of data we then need is π(*a*). We use the total population (both sexes combined) by five-year age groups for each country for the years 1990–2015. The data are available from the United Census Bureau (https://www.census.gov/population/international/data/idb/region.php).

Next, based on the health stock data, global and regional health inequalities in health stock were measured using the Gini coefficient. The Gini coefficient or absolute Gini index is defined as twice the area between the Lorenz curve and the diagonal line, multiplied by the mean value of the variable of interest [27]. In this study, we measured the health stock Gini coefficient based on the true Lorenz curve as applied by Le Grand (1987). The Gini coefficient was calculated for each year based on each country ranked from the worst to the best level of the variable and weighted by the size of the population of the country. The Gini coefficient ranges from 0 to 1, which represents perfect equality, with 1 indicating perfect inequality [28], using the following formula:

$$ G=2\sum \limits_{t-1}^T{\mu}_t\times {f}_t\times {R}_t-{\mu}_{,} $$

where G is the Gini coefficient, μ is the mean value of the variable, T is the number of countries (140 countries), μ_t_ is the value of the variable (health stock) in the t^th^ country, f_t_ is the country’s population share, and R_t_ is the relative rank of the t^th^ country ranked from worst to best level of health stock.

We explain data sources and other details in our analysis of inequality and national wealth. The publicly available data on GDP, working-age population from ages 15 to 64, mortality rate, fertility rate, life expectancy and total population were retrieved from the World Bank online open data database (https://data.worldbank.org). Inclusive wealth data (1990–2014) were obtained from Managi and Kumar [18]. The net income of the firm-level data from countries that had survived for more than 100 years and already achieved sustainability was taken from Oshika and Saka [29] to analyze the trend in health stock. However, due to data limitations, out of 136 countries, only 43 were used for analysis as a proxy of the firm’s sustainability from 1990 to 2010. Last, Pearson’s correlation coefficient was used to measure the relationship between growth in health stock and national wealth (GDP, IWI and firm-level net income).

## Results

### Global health stock inequality (1990–2015)

**Fig. 1:**
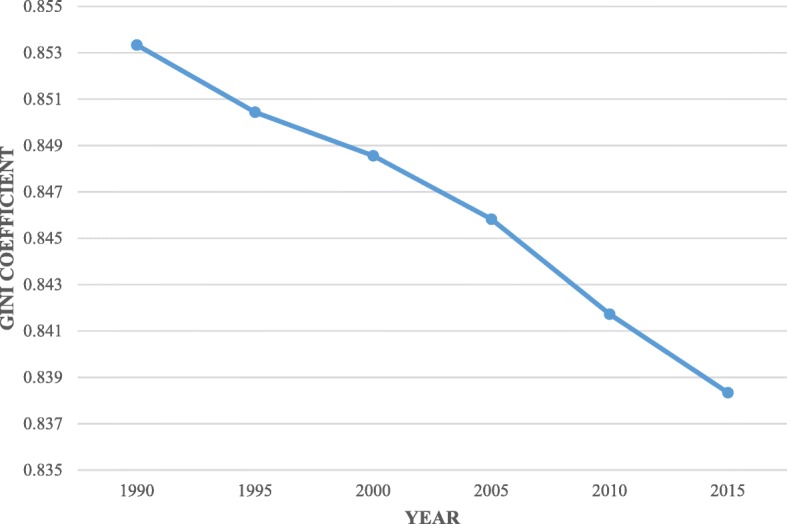
Gini coefficient of health stock among 140 countries (1990–2015)

The overall trend in health inequalities in terms of health stock declined globally from 1990 to 2015, as illustrated in Fig. 1. The increase in global life expectancy helped reduce global health inequalities [30]. Moreover, on the global average, the trend in life expectancy at birth has risen steadily over time [31]. Social factors such as economic growth, technology, reduced inequalities, knowledge of and investment in public health and health systems are also contributed to the decline in global health inequality [32].

However, the average Gini coefficient (0.846) indicates that inequality in global health stock remains relatively widespread (Fig. 1). This is because even though enormous improvements in health have been made in the last century, particularly in its last half (from the 1950s to 2000s), developing countries benefited unequally. These countries continue to have high mortality rates, while some countries are plagued with the burden of ill health, including infectious and parasitic diseases [33]. Furthermore, although the health conditions of the population generally showed a positive trend globally, healthcare inequalities still exist, especially in poor and developing countries. In many cases, inequalities have spread between countries or between regions and social or ethnic groups within the same nation. For example, it is reported that most of nearly 104 million children aged 6 to 59 months worldwide were underweight in 2010, and the majority of these (65 million children) lived in Sub-Sahara Africa (SSA) and South Asia [34]. These results are also consistent with a study by the Global Burden of Diseases, Injuries, and Risk Factors Study (GBD) 2015 Healthcare Access and Quality Collaborators [35]. That study, based on the Healthcare Access and Quality Index (HAQ Index), showed that nearly all countries and territories saw their HAQ Index values improve; nonetheless, the difference between the highest and lowest observed HAQ Index was larger in 2015 than in 1990 [35].

### Regional and income group

Table 1 lists the changes in the Gini coefficient by region. All countries were categorized into six regions: Africa, Asia, Europe, Latin America and the Caribbean (LAC), North America and Oceania. The average Gini coefficient across 140 countries over the 25 years was 0.846. Europe was the region with the highest average Gini coefficient over the sample period, at 0.849. This indicates that European countries face health inequalities within their population. At the start of the twenty-first century, all European countries faced substantial inequalities in health within their population [36]. Thus, under Health 2020, the European health policy framework and strategy for the twenty-first century aims to improve health for all and reduce health inequalities through improved leadership and governance of health [37].

**Table 1:** Gini coefficient of health stock in 1990 and 2015, and change over time by regions

Regions	Number of countries	1990	2015	Change 1990–2015	Average 1990–2015
Global	140	0.853	0.846	−0.007	0.846
High-Income Countries (HICs)	42	0.781	0.769	−0.012	0.776
Upper Middle Income Countries (UMICs)	35	0.871	0.863	−0.008	0.866
Lower Middle Income Countries (LMICs)	37	0.871	0.866	−0.005	0.841
Low Income Countries (LICs)	26	0.692	0.660	−0.032	0.675
Africa	37	0.859	0.834	−0.025	0.846
Asian	37	0.782	0.762	−0.020	0.772
Europe	35	0.848	0.851	0.003	0.849
Latin America and Caribbean (LAC)	25	0.565	0.593	0.028	0.567
North America	2	0.054	0.031	−0.023	0.043
Oceania	4	0.739	0.766	0.027	0.749

According to Table 1 above, LAC showed the highest percentage growth of the Gini coefficient by 2.8% from 1990 to 2015. Based on the analysis (see Table 2), LAC’s working-age population was the second largest (an increase of 72.4%) after Africa (an increase of 100%) from 1990 to 2014, which could be one of the factors contributing to the highest percentage growth of the Gini coefficient. In addition, there are three different country profiles in the LAC region [38]. The population of the first group of countries was considered young at the initial phase of the demographic and epidemiological transition, including countries such as Bolivia, Guatemala, Haiti and Honduras. The second group of countries, including Brazil, Colombia, Peru and the Dominican Republic, were at an intermediate level of transition, where the total fertility rate and the death rates were declining, but still had a large proportion of the young population. The third group of countries included aged societies, advanced in the demographic transition, with fertility rates below replacement levels, such as Cuba, Uruguay and Costa Rica. Overall, in the sample period, most LACs had a large proportion of youths contributing to the working-age population (aged 15 to 64).

**Table 2:** Median percentage growth (%) (1990–2014)

Regions	Health Stock	GDP	IWI	Working age population	Mortality Rate	Fertility rate	Life expectancy at birth	Total population
Worldwide	30.6	120.9	61.1	50.1	−62.1	−35.5	8.4	27.6
Africa	84.2	192.3	36	100	−45.9	−23.3	8.9	92.3
Asia	62.4	273.6	73.5	67.9	−67.0	−44.7	8.4	60.8
Europe	19.1	34.5	38.7	5.5	−63.4	−16.4	7.6	23.2
Latin America and the Caribbean (LAC)	53.1	199.1	102.6	72.4	−60.3	−32.7	8.7	51.7
Northern America	26.7	78.2	46.1	28.9	−35.8	−11.9	5.2	26.1
Oceania	55.3	96.3	24.5	66	−26.7	−19.6	7.3	59.8

**Fig. 2:**
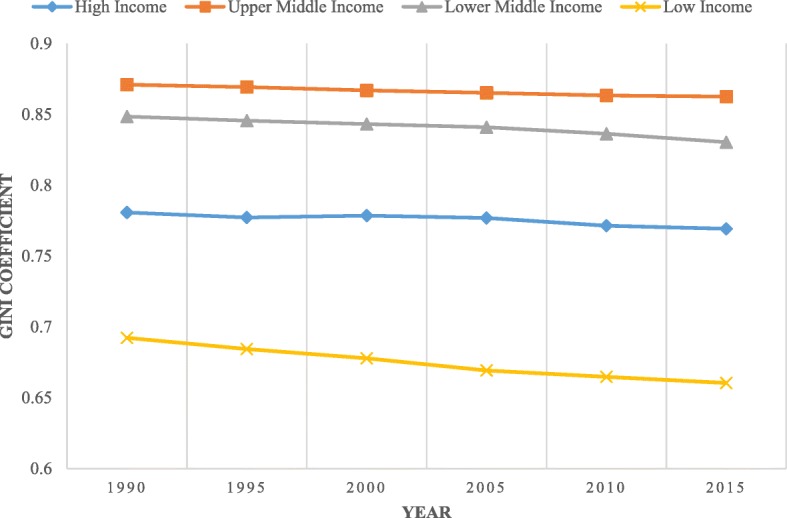
Gini coefficient categorized by income groups (1990–2015)

**Fig. 3:**
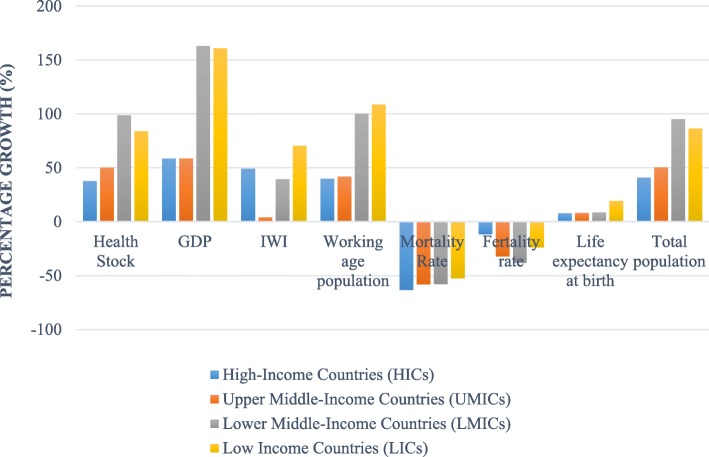
Median percentage growth by regions and income groups (1990–2014)

Overall, in most groups by income countries, inequalities have decreased. Figure 2 shows that the Gini coefficient of health stock declined rapidly from 1990 to 2015, especially for LICs, and declined slowly in lower-middle income countries (LMICs) and upper-middle income countries (UMICs). The Gini coefficient for LICs indicated steadily decline, from 0.69 in 1990 to 0.66 in 2015. From the analysis (see Fig. 3), the working-age population (an increase of 108.6%) and growth in life expectancy at birth (an increase of 19.2%) in LICs were most likely the contributing factors to the decline in inequality. LICs, particularly for SSA, have the world’s highest youth population growth rate (nearly 20% of SSA’s total population) and the highest share of youth in the working-age population [39]. SSA alone is likely to account for nearly two-thirds of the growth in the world’s working-age population between 2015 and 2050 [40].

**Fig. 4:**
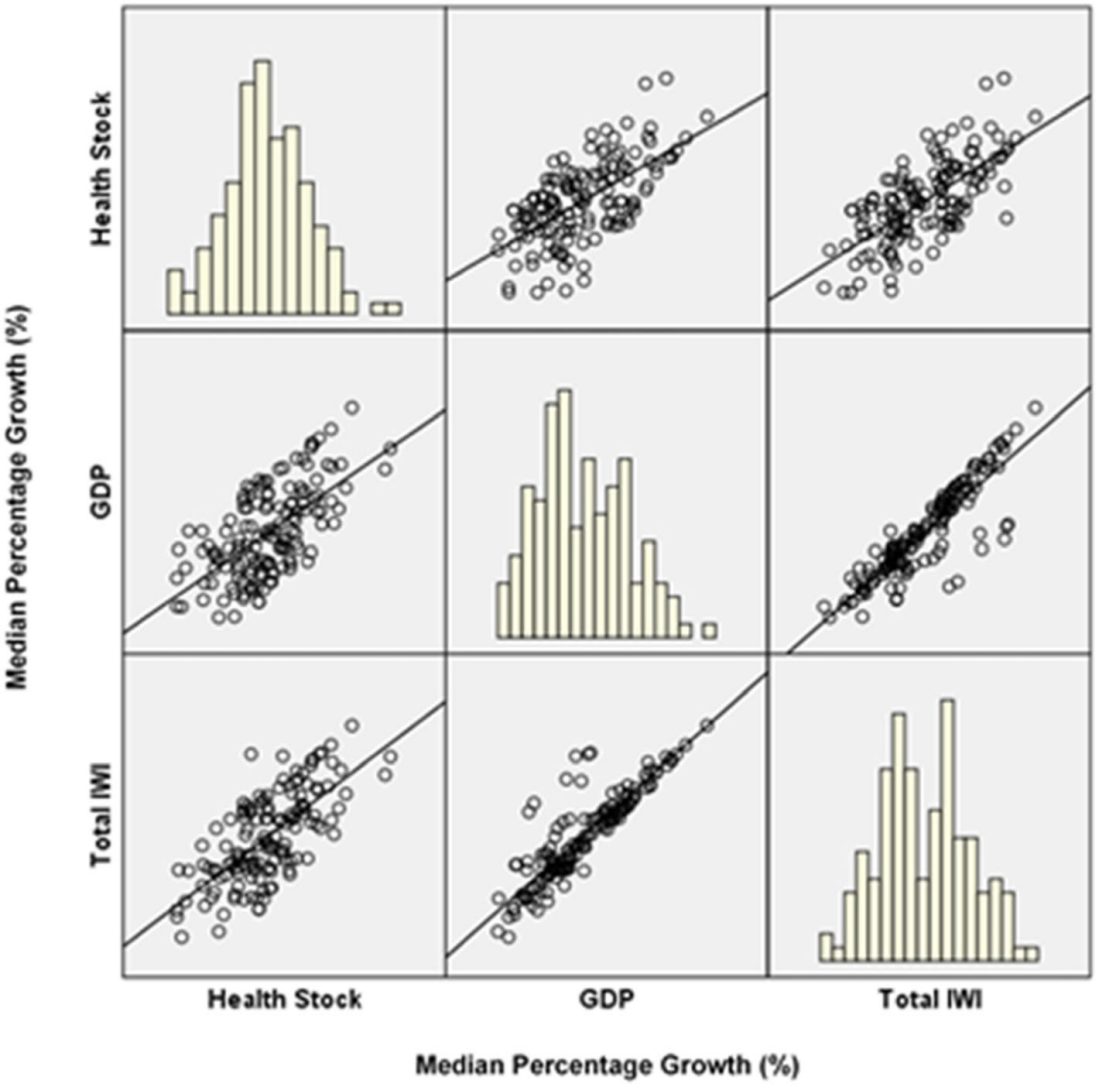
Scatterplot matrix median health stock, GDP and total inclusive wealth, 140 countries

Although fertility trends showed that SSA experienced an extremely slow decline in fertility compared to other regions of the world, SSA fertility rates remain high, which could lead to high youth dependency [41, 42]. With more than one-third of the total population aged 10 to 24, this large number of young people represents an opportunity to accelerate economic growth and reduce poverty. SSA could also obtain benefits from a significant demographic dividend if SSA made the right investments in the current and future generation. Furthermore, according to World Health Statistics 2014, LICs have made the greatest progress, with an average increase in life expectancy of 9 years from 1990 to 2012 [43].

### Association among inequality and other statistics

**Fig. 5:**
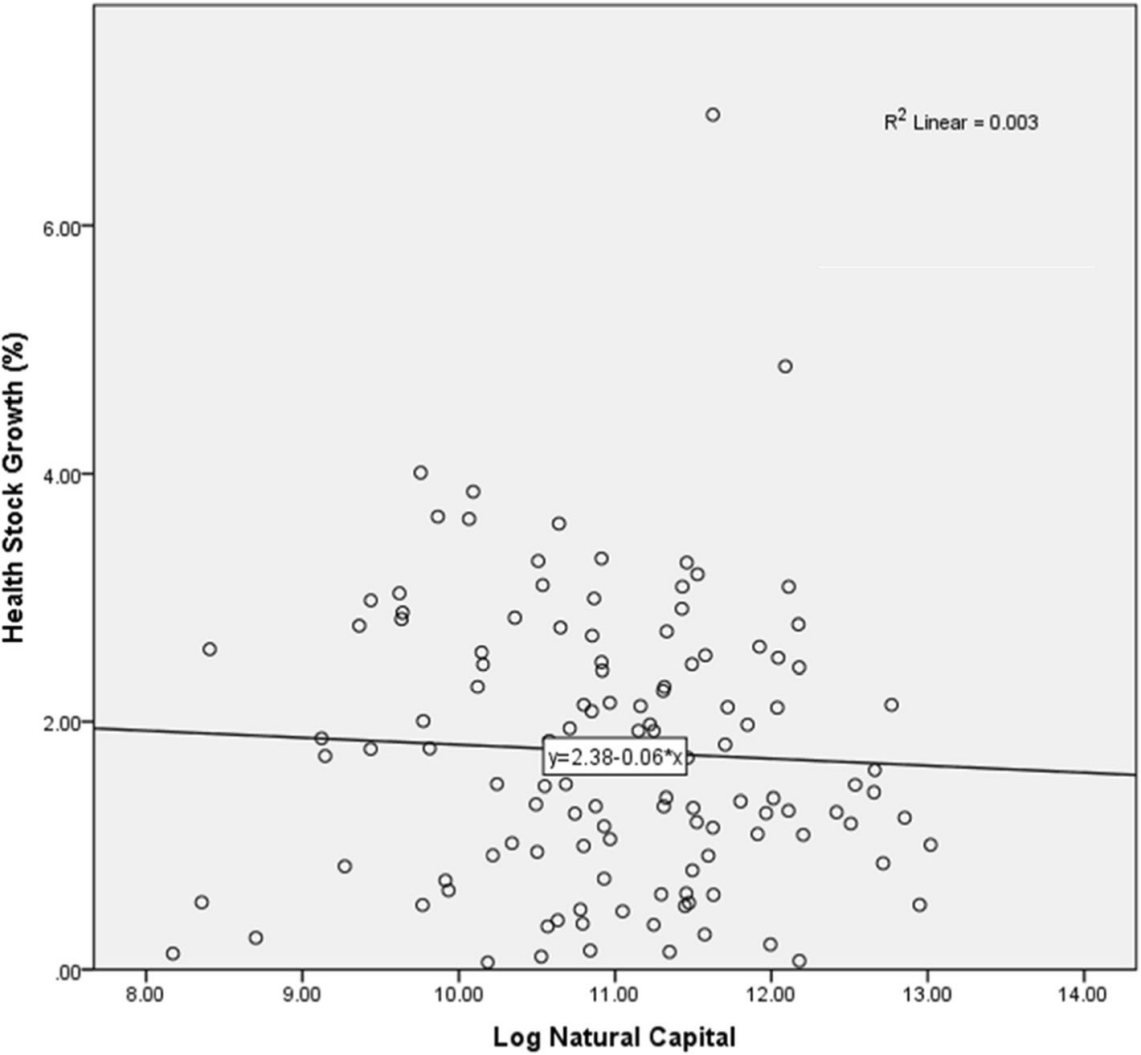
Relationship between health stock growth (%) and log natural capital, 140 countries

In this study, we also measure the relationship between health stock growths and other national wealth statistics. Referring to Table 3 (see Appendix) and Fig. 4, most countries showing positive growth in health stock also saw a positive relationship with the GDP and IWI. This indicates that the country’s growing health stock influences the growth of GDP and IWI. Previous studies have shown that an increase in health is associated with rising national wealth. For instance, the world has experienced impressive improvements in wealth and health, the world’s real GDP per capita having increased by 180% from 1970 to 2007 and accompanied by a 50% decline in the infant mortality rate [44]. On the other hand, the expectation for the relationship between a country’s wealth and health is affected by economic growth, which improves the population’s health [45, 46]. Results in this study show that health stock also contributed to a strong positive relationship with GDP and IWI, or vice versa. We should note that some countries did show a negative relationship between health stock growth and natural capital as part of IWI (Fig. 5), although their GDP yielded a strong relationship with health stock.

**Fig. 6:**
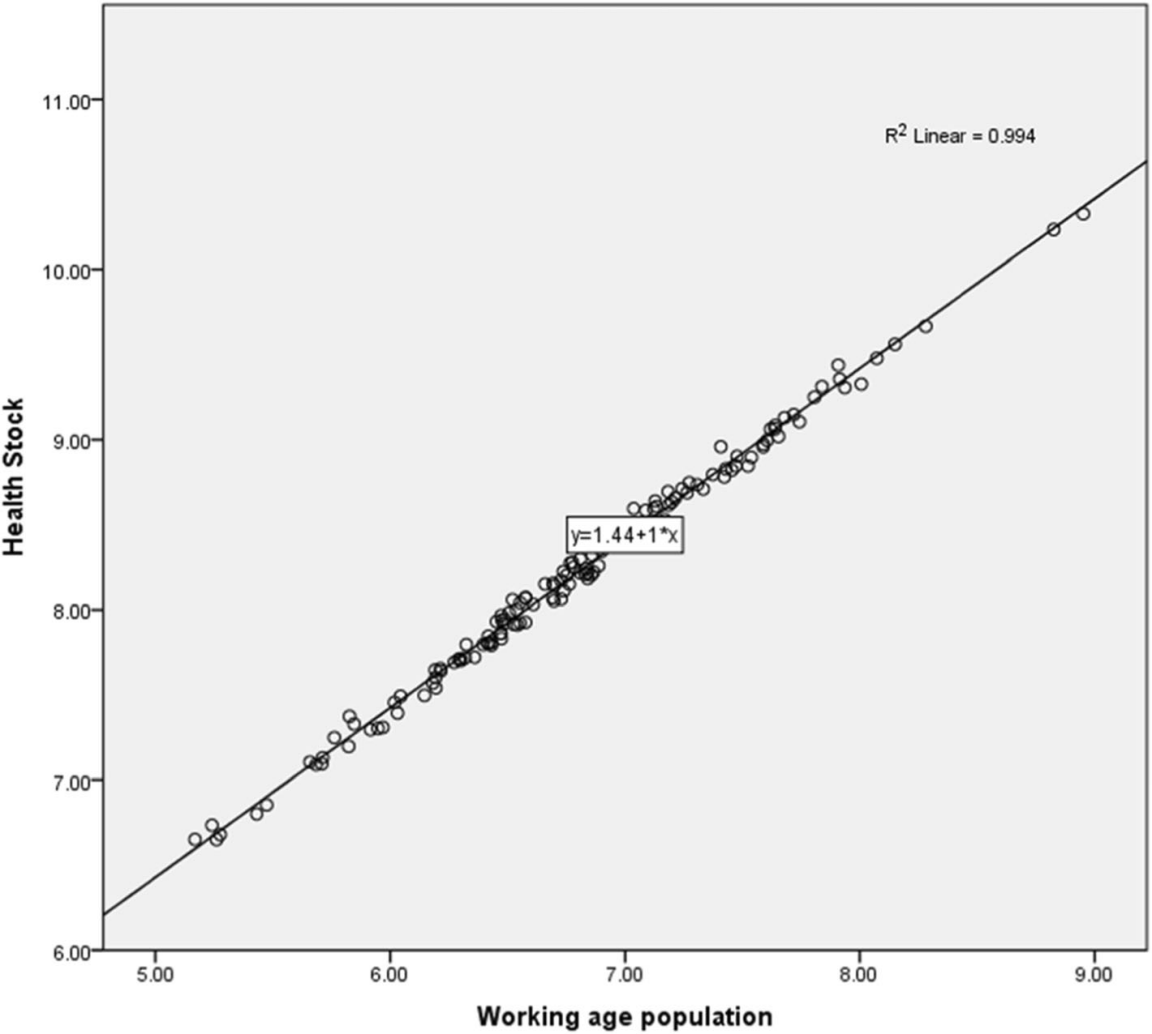
Relationship between health stock and working-age population, 140 countries

Last, we try to investigate the main factors contributing to the growth of health stock. Countries with greater health stock had a strong positive relationship with the working-age population (see Fig. 6). Therefore, growth in the working-age population had a positive impact on health stock or vice versa. On this point, several studies showed that a change in the amount of working-age population can have a large impact on economic growth. For instance, the aging population in countries such as Japan means that a relatively smaller cohort of the working-age population will slow Japan’s economic growth unless there is a substantial rise in productivity and per capita output [47]. In East Asia, the shift in age structure resulted in the working-age population growing between 1965 and 1990 at nearly 10 times the rate of the dependent population, contributing to rapid economic growth. These countries successfully shifted their age structures to obtain a boost in economic productivity, known as the demographic dividend [48]. In 1990, 67% of the population in East Asia comprised the working-age population. With an increase in working-age share, countries stand to benefit from a proportional increase in the pool of potential workers in the economy, and thus per capita income can increase [49, 50].

**Fig. 7:**
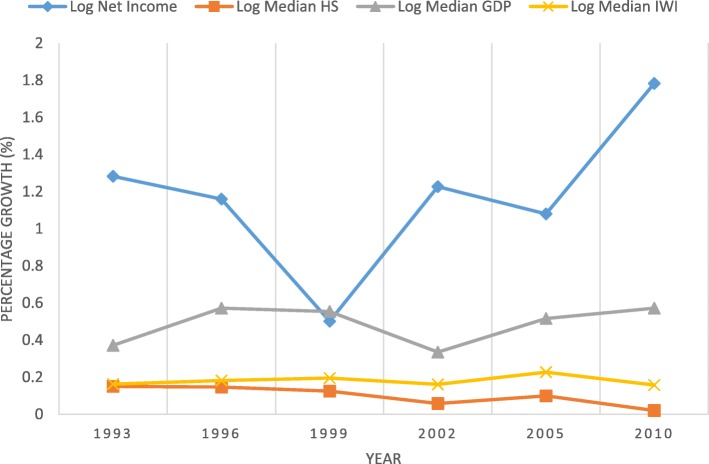
Log of median growth of net income (firm-level), GDP, IWI and health stock, 43 countries (1990–2010)

Based on firm-level analysis (Fig. 7), we found that countries’ trend of median net income growth was not influenced by the median growth of health stock from 1990 to 2010; for instance, in 1996 and 2002, even though the firm’s median net income showed increases 1.16 and 1.22% respectively. At that time, however, median health stock, GDP and IWI showed no obvious changes, although the decrease in the line of Log Net Income in 1999 was due to temporary world economic crises caused by the Asian and Russian Currency Crises.

## Discussion

In this study, we used stock-based health capital and the Gini coefficient to measure health inequality. We presented global health stock inequalities for 140 countries from 1990 to 2015. We also analyzed the inequality of health stock in relation to national wealth (both GDP and IWI) and provided a firm-level net income analysis. Based on our analyses, even though relative global health inequality declined from 1990 to 2015, the average Gini coefficient indicates that inequality in global health stock is still relatively widespread. This result shows that measuring health stock using the capital approach and the Gini coefficient can be used to reveal health inequality. The median health stock increased by 30%, and 122 of 140 countries representing 86.4% of our sample showed increases in health stock growth between 1990 and 2015. The results also showed that health stock contributed to a strong positive relationship with GDP and IWI. However, some countries indicated a negative relationship between health stock and natural capital, which is part of IWI, even though the GDP of those countries yielded a strong positive relationship with health stock. A negative relationship is a sign of unsustainable development because sustainable development involves not only maintaining GDP but also IWI, as it is a collective set of assets comprising human, produced and natural capital [8, 51]. In particular, we can confirm the importance of monitoring changes in health stock from 12 countries which showed a negative relationship of health stock to both GDP and IWI: Poland, Slovenia, Japan, Hungary, Guyana, Romania, the Russian Federation, Albania, Estonia, Bulgaria, Lithuania, and Latvia (see Appendix). These findings support the usefulness of health stock and IWI as important components of and indicators for judging national sustainability. Once economists and researchers in the social sciences recognized the problems involved in using GDP as the sole measure of well-being or economic welfare, alternative policy-making measures were developed and promoted starting in the early 1970s [52]. Therefore, health stock should be consistently included in IWI, which could complement GDP as an important instrument to measure progress toward sustainable development.

In a detailed regional analysis, the Gini coefficient of health stock declined rapidly from 1990 to 2015, especially for LICs, and declined slowly in LMICs and UMICs. Based on our analysis, rapid population growth and a higher share of youth in the working-age population of LICs were most likely contributing factors to the declining inequality. However, all this growth can generate a virtuous circle of prosperity and opportunity if all parties work together on the improvement, development and implementation of a policy coherent with sustainability development. For instance, LICs can draw from the Asian Tigers in terms of how to harness the demographic dividend for accelerated economic growth and social transformations and achieve the SDGs.

This study also examined the impact of health stock on net income at the firm level. We found that firms that have already achieved sustainability are not influenced by a country’s health stock but probably by other factors, e.g., innovations, human resources (employees), organizational culture and strategy. TenHaken [53] posited that three common characteristics have enabled long-lived business firms in Japan and the United States to overcome environmental changes and economic challenges and to prosper for long periods of time: first, the clarity and continuity of corporate culture and values; second, learning systems built on relationships, especially with their customers and suppliers; and third, the art of balancing tradition and innovation. In addition, Napolitano et al. [54] argue that internal characteristics, management practices, and strategic decisions are also factors furthering business longevity. Moreover, Japanese and German firms recognize that organizational culture is one of the crucial factors for a company’s longevity [55]. Thus, it can be concluded that firm-level net income is not influenced by a country’s health stock.

Although our primary objective was to clarify the relationship between inequality of health stock and national wealth, our study has two main limitations: a causality issue and an issue related to data availability. First, we didn’t explicitly identify the causal relationship between health stock and national wealth. When we consider the association between stock-based indicators and flow-based ones, such as GDP, the causality direction is relatively clear because flow outputs are produced from the amount of capital stock, based on production framework in economics [8, 10, 11]. However, we should carefully identify the causality if we focus on explicitly uncovering the causal relationship between stock-based indicators. Second, the health stock indicator we used in this study doesn’t include the effects of illness and injury on a healthy life, but only captures the aspects of longevity. We could use a quantitative database of illness and injury, such as GBD [35], to quantify the amount of stock decreased by each disease, although it would require significant effort to estimate the shadow price (cost) of disease. Despite the above two limitations, the expected modifications are beyond the scope of this research and we leave them for future research.

## Conclusions

In this study, we investigated the associations between inequality of health stock and the various indices related to national wealth. Although we used relatively simple statistical methods, we demonstrated that health stock was an important component in IWI for judging health-related sustainability. As a policy implication, measuring health stock in relation to national wealth not only helps in achieving SDG 3 but also SDG 10, which calls for a reduction in national inequalities. Beyond GDP, we conclude that IWI, which includes the values of health stock, is more comprehensive in measuring national wealth and can be used to complement GDP in tracking sustainability.

## Data Availability

The datasets used and/ or analysed during the current study are available from the corresponding author on reasonable request.

## Appendix

**Table 3:** Summary of Pearson correlations between health stock with GDP, IWI, Produce Capital (PC), Human Capital (HC), Natural Capital (NC), life expectancy, fertility rate, mortality rate, population and working-age population

Country	Health stock growth (%)	*Pearson Correlation*									
		GDP	IWI	PC	HC	NC	Life expectancy	Fertility Rate	Mortality rate	Total population	Working age population
Qatar	6.9	.997^a^	.984^a^	.976^a^	.993^a^	−.987^a^	.925^a^	−.934^a^	−.806^a^	.996^a^	.994^a^
United Arab Emirates	4.9	.992^a^	.955^a^	.958^a^	.959^a^	−.999^a^	.989^a^	−.944^a^	−.964^a^	.970^a^	.961^a^
Bahrain	4.0	.995^a^	.976^a^	.986^a^	.973^a^	−.945^a^	.950^a^	−.928^a^	−.865^a^	.997^a^	.997^a^
Afghanistan	3.9	.795^a^	.971^a^	.596^a^	.979^a^	−.923^a^	.992^a^	−.856^a^	−.995^a^	.990^a^	.986^a^
Jordan	3.7	.984^a^	.994^a^	.990^a^	.994^a^	−.945^a^	.980^a^	−.880^a^	−.977^a^	.990^a^	.990^a^
Niger	3.6	.989^a^	.988^a^	.850^a^	.999^a^	−.840^a^	.992^a^	−.990^a^	−.981^a^	1.000^a^	.999^a^
Uganda	3.6	.997^a^	.997^a^	.984^a^	.998^a^	−.991^a^	.982^a^	−.993^a^	−.970^a^	.999^a^	1.000^a^
Yemen	3.3	.959^a^	.994^a^	.981^a^	.999^a^	−0.34479464	.999^a^	−.982^a^	−.984^a^	.999^a^	1.000^a^
Benin	3.3	.997^a^	.994^a^	.991^a^	.999^a^	−.958^a^	.982^a^	−.987^a^	−.990^a^	1.000^a^	1.000^a^
Congo	3.3	.986^a^	.977^a^	.931^a^	.992^a^	−.962^a^	.844^a^	−.952^a^	−.848^a^	.994^a^	.991^a^
Zambia	3.2	.980^a^	.992^a^	.900^a^	.996^a^	−.971^a^	.975^a^	−.998^a^	−.974^a^	.996^a^	.996^a^
Liberia	3.1	.961^a^	−0.14548	.652^a^	.983^a^	−.963^a^	.982^a^	−.985^a^	−.975^a^	.995^a^	.993^a^
United Republic of Tanzania	3.1	.993^a^	.998^a^	.983^a^	.998^a^	0.28074611	.984^a^	−.963^a^	−.971^a^	.999^a^	.999^a^
Democratic Republic of the Congo	3.1	.499^b^	.983^a^	−0.1145	.998^a^	−.996^a^	.975^a^	−.896^a^	−.997^a^	.999^a^	.999^a^
Gambia	3.0	.986^a^	.888^a^	.986^a^	.985^a^	−.719^a^	.997^a^	−.989^a^	−.996^a^	.994^a^	.993^a^
Mali	3.0	.997^a^	.943^a^	.998^a^	.999^a^	−.822^a^	.991^a^	−.988^a^	−.992^a^	1.000^a^	1.000^a^
Rwanda	3.0	.974^a^	.984^a^	.916^a^	.988^a^	.783^a^	.942^a^	−.932^a^	−.930^a^	.988^a^	.984^a^
Mozambique	2.9	.975^a^	.835^a^	.881^a^	.992^a^	.679^a^	.996^a^	−.995^a^	−.985^a^	.993^a^	.996^a^
Burundi	2.9	.842^a^	.991^a^	.926^a^	.994^a^	−.600^a^	.976^a^	−.996^a^	−.995^a^	.997^a^	.994^a^
Malawi	2.8	.988^a^	.995^a^	.957^a^	.989^a^	−.906^a^	.986^a^	−.975^a^	−.936^a^	.985^a^	.993^a^
Togo	2.8	.952^a^	.994^a^	.674^a^	.997^a^	−.872^a^	.685^a^	−.973^a^	−.992^a^	.999^a^	.997^a^
Iraq	2.8	.955^a^	.978^a^	.780^a^	.996^a^	−.993^a^	.533^a^	−.891^a^	−.989^a^	.996^a^	.997^a^
Maldives	2.8	.955^a^	.935^a^	.942^a^	.985^a^	−.730^a^	.983^a^	−.917^a^	−.983^a^	.976^a^	.972^a^
Kenya	2.8	.991^a^	.993^a^	.985^a^	.988^a^	−0.161213874	.839^a^	−.983^a^	−.974^a^	.990^a^	.989^a^
Cameroon	2.7	.987^a^	.440^b^	.980^a^	.998^a^	−.852^a^	.879^a^	−.967^a^	−.888^a^	1.000^a^	.999^a^
Senegal	2.7	.996^a^	.982^a^	.993^a^	.998^a^	−.746^a^	.965^a^	−.924^a^	−.970^a^	1.000^a^	1.000^a^
Nigeria	2.6	.983^a^	.976^a^	.716^a^	.996^a^	−.997^a^	.970^a^	−.985^a^	−.995^a^	1.000^a^	.999^a^
Singapore	2.6	.984^a^	.993^a^	.992^a^	.993^a^	−.549^a^	.997^a^	−.938^a^	−.908^a^	.999^a^	.989^a^
Mauritania	2.6	.976^a^	.964^a^	.886^a^	.998^a^	−.615^a^	.995^a^	−.989^a^	−.908^a^	.999^a^	.999^a^
Sudan (former)	2.5	.951^a^	.979^a^	.896^a^	.979^a^	−.755^a^	.974^a^	−.994^a^	−.989^a^	.987^a^	.986^a^
Gabon	2.5	.818^a^	.950^a^	.971^a^	.995^a^	−.966^a^	.617^a^	−.968^a^	−.991^a^	.995^a^	.989^a^
Honduras	2.5	.984^a^	.981^a^	.995^a^	.994^a^	−.995^a^	.983^a^	−1.000^a^	−.992^a^	1.000^a^	.995^a^
Papua New Guinea	2.5	.907^a^	.461^b^	.901^a^	.995^a^	0.201209992	.996^a^	−.988^a^	−.986^a^	.999^a^	.998^a^
Israel	2.5	.993^a^	.997^a^	.997^a^	.996^a^	−.952^a^	.994^a^	.772^a^	−.983^a^	.999^a^	.999^a^
Kuwait	2.4	.909^a^	.626^a^	.839^a^	.840^a^	−.908^a^	.892^a^	−.755^a^	−.876^a^	.833^a^	.841^a^
Côte d’Ivoire	2.4	.926^a^	.996^a^	−.829^a^	.998^a^	.821^a^	0.092503004	−.991^a^	−.972^a^	.998^a^	.996^a^
Belize	2.3	.992^a^	.993^a^	.995^a^	.993^a^	−.987^a^	−0.17016075	−.990^a^	−.953^a^	.999^a^	.997^a^
Central African Republic	2.3	.527^a^	−.892^a^	−0.30506	.998^a^	−.985^a^	0.300921174	−.997^a^	−.960^a^	.976^a^	.960^a^
Cambodia	2.2	.958^a^	−.703^a^	.918^a^	.997^a^	−.982^a^	.978^a^	−.983^a^	−.931^a^	.997^a^	.987^a^
Guatemala	2.2	.998^a^	.994^a^	.996^a^	.996^a^	−.965^a^	.988^a^	−.996^a^	−.982^a^	.999^a^	.994^a^
Botswana	2.1	.980^a^	.988^a^	.987^a^	.979^a^	−.958^a^	.481^b^	−.838^a^	−.738^a^	.955^a^	.952^a^
Saudi Arabia	2.1	.916^a^	.970^a^	.882^a^	.979^a^	−.734^a^	.978^a^	−.993^a^	−.965^a^	.980^a^	.969^a^
Lao People’s Democratic Republic	2.1	.960^a^	.814^a^	.940^a^	.995^a^	−.439^b^	.998^a^	−.988^a^	−.998^a^	1.000^a^	.990^a^
Malaysia	2.1	.983^a^	.991^a^	.990^a^	.993^a^	−.981^a^	.996^a^	−.985^a^	−.946^a^	.999^a^	.996^a^
Pakistan	2.1	.981^a^	.983^a^	.996^a^	.993^a^	−.999^a^	.998^a^	−.983^a^	−.999^a^	.997^a^	.994^a^
Ghana	2.1	.978^a^	.985^a^	.949^a^	.999^a^	−0.379000818	.943^a^	−.969^a^	−.992^a^	.998^a^	.999^a^
Haiti	2.0	.709^a^	.914^a^	.911^a^	.916^a^	−.927^a^	.921^a^	−.939^a^	−.994^a^	.933^a^	.928^a^
Egypt	2.0	.994^a^	.998^a^	.997^a^	.996^a^	−.987^a^	.933^a^	−.646^a^	−.934^a^	.996^a^	.989^a^
Bolivia (Plurinational State of)	2.0	.981^a^	0.28263	.983^a^	.996^a^	−.998^a^	.998^a^	−.997^a^	−.990^a^	.999^a^	.999^a^
Namibia	1.9	.992^a^	.984^a^	.958^a^	.994^a^	.695^a^	−0.104628892	−.905^a^	−.855^a^	.984^a^	.987^a^
Philippines	1.9	.973^a^	.996^a^	.992^a^	.997^a^	.466^b^	.987^a^	−.997^a^	−.972^a^	.999^a^	.999^a^
Nepal	1.9	.974^a^	0.069673	.975^a^	.987^a^	−.962^a^	.999^a^	−.987^a^	−.998^a^	1.000^a^	.993^a^
Cyprus	1.9	.953^a^	.996^a^	.992^a^	.995^a^	−0.052281633	.998^a^	−.955^a^	−.985^a^	.997^a^	.995^a^
Nicaragua	1.8	.960^a^	.962^a^	.915^a^	.982^a^	−.954^a^	.998^a^	−.990^a^	−.996^a^	.994^a^	.987^a^
Algeria	1.8	.975^a^	.996^a^	.943^a^	.995^a^	−.996^a^	.987^a^	−.644^a^	−.991^a^	1.000^a^	.992^a^
Costa Rica	1.8	.972^a^	.981^a^	.957^a^	.995^a^	0.188450467	.998^a^	−.993^a^	−.975^a^	.999^a^	.997^a^
Paraguay	1.8	.926^a^	.996^a^	.971^a^	.993^a^	−.907^a^	.997^a^	−.997^a^	−1.000^a^	1.000^a^	.996^a^
Tajikistan	1.8	.535^a^	.994^a^	−.968^a^	.996^a^	−.534^a^	.990^a^	−.885^a^	−.962^a^	.999^a^	.996^a^
Swaziland	1.8	.990^a^	.974^a^	.673^a^	.988^a^	.982^a^	−0.298820066	−.933^a^	0.084098499	.978^a^	.988^a^
Luxembourg	1.7	.966^a^	.998^a^	.995^a^	.993^a^	.689^a^	.989^a^	−.675^a^	−.914^a^	.999^a^	.994^a^
Ecuador	1.7	.962^a^	.982^a^	.948^a^	.996^a^	−.994^a^	.985^a^	−.979^a^	−.975^a^	.999^a^	.999^a^
Panama	1.6	.945^a^	.978^a^	.931^a^	.998^a^	−.431^b^	.999^a^	−.965^a^	−.995^a^	.999^a^	1.000^a^
Venezuela (Bolivarian Republic of)	1.6	.897^a^	.963^a^	.894^a^	.996^a^	−.999^a^	.986^a^	−.989^a^	−.977^a^	1.000^a^	.999^a^
Sierra Leone	1.5	.877^a^	.968^a^	.806^a^	.978^a^	−.895^a^	.965^a^	−.948^a^	−.947^a^	.969^a^	.971^a^
Bangladesh	1.5	.947^a^	.975^a^	.927^a^	.988^a^	−.614^a^	1.000^a^	−.997^a^	−1.000^a^	.999^a^	.997^a^
India	1.5	.963^a^	.985^a^	.943^a^	.997^a^	−.988^a^	.997^a^	−.998^a^	−.999^a^	.999^a^	.999^a^
Dominican Republic	1.5	.990^a^	.996^a^	.991^a^	.996^a^	−.955^a^	.996^a^	−.985^a^	−.961^a^	.999^a^	.999^a^
Iran (Islamic Republic of)	1.4	.954^a^	.983^a^	.950^a^	.988^a^	−.982^a^	.995^a^	−.907^a^	−.990^a^	.995^a^	.982^a^
Mongolia	1.4	.930^a^	0.040494	.905^a^	.994^a^	−.867^a^	.995^a^	−0.376136714	−.982^a^	.996^a^	.992^a^
Mexico	1.4	.989^a^	.992^a^	.987^a^	.995^a^	−.986^a^	.985^a^	−.981^a^	−.982^a^	.999^a^	.998^a^
Turkey	1.4	.970^a^	.992^a^	.980^a^	.994^a^	−.971^a^	.995^a^	−.986^a^	−.994^a^	.998^a^	.999^a^
Ireland	1.3	.920^a^	.992^a^	.996^a^	.988^a^	−0.303411632	.990^a^	0.338273565	−.968^a^	.996^a^	.956^a^
Morocco	1.3	.964^a^	.989^a^	.958^a^	.996^a^	−0.123423879	.994^a^	−.904^a^	−.995^a^	.998^a^	.999^a^
Myanmar	1.3	.926^a^	−0.21738	.802^a^	.993^a^	−.992^a^	.996^a^	−.979^a^	−.996^a^	.997^a^	.997^a^
Viet Nam	1.3	.974^a^	.999^a^	.944^a^	.999^a^	−.622^a^	.999^a^	−.869^a^	−.972^a^	.999^a^	.997^a^
Peru	1.3	.943^a^	.927^a^	.899^a^	.991^a^	−.752^a^	.995^a^	−.983^a^	−.989^a^	.998^a^	.996^a^
Indonesia	1.3	.937^a^	.986^a^	.977^a^	.994^a^	−.992^a^	.997^a^	−.866^a^	−.998^a^	.996^a^	.999^a^
Colombia	1.3	.963^a^	.979^a^	.958^a^	.997^a^	−.745^a^	.994^a^	−.992^a^	−.995^a^	.998^a^	.998^a^
Syrian Arab Republic	1.3	.949^a^	.955^a^	.876^a^	.955^a^	−.919^a^	0.356648404	−.913^a^	−.937^a^	.969^a^	.972^a^
Brazil	1.2	.963^a^	.983^a^	.950^a^	.992^a^	−.998^a^	1.000^a^	−.996^a^	−.995^a^	1.000^a^	.999^a^
Chile	1.2	.978^a^	.972^a^	.944^a^	.985^a^	−.915^a^	.998^a^	−.998^a^	−.948^a^	.996^a^	.992^a^
Australia	1.2	.998^a^	.985^a^	.975^a^	.989^a^	−.972^a^	.993^a^	0.314032154	−.971^a^	.989^a^	.993^a^
Zimbabwe	1.2	.923^a^	.592^a^	0.258223	.547^a^	−.501^b^	.686^a^	−0.204894318	−.803^a^	.599^a^	.555^a^
South Africa	1.1	.968^a^	.983^a^	.985^a^	.959^a^	−.969^a^	− 0.326603591	−.868^a^	−.647^a^	.962^a^	.955^a^
Argentina	1.1	.935^a^	.992^a^	.958^a^	.998^a^	−.842^a^	.997^a^	−.970^a^	−.985^a^	1.000^a^	1.000^a^
New Zealand	1.1	.992^a^	.921^a^	.984^a^	.997^a^	.614^a^	.990^a^	0.031606767	−.952^a^	.999^a^	.996^a^
Iceland	1.1	.982^a^	.829^a^	.992^a^	.985^a^	−.659^a^	.962^a^	−0.370058434	−.966^a^	.993^a^	.999^a^
Tunisia	1.0	.979^a^	.989^a^	.974^a^	.991^a^	−.996^a^	.986^a^	−.827^a^	−.995^a^	.998^a^	.998^a^
United States of America	1.0	.990^a^	.995^a^	.994^a^	.994^a^	−.982^a^	.992^a^	−.447^b^	−.974^a^	.999^a^	.999^a^
Sri Lanka	1.0	.948^a^	.982^a^	.941^a^	.982^a^	−.916^a^	.954^a^	−.836^a^	−.978^a^	.995^a^	.986^a^
Jamaica	0.9	.852^a^	.996^a^	.994^a^	.991^a^	−.788^a^	.928^a^	−.997^a^	−.991^a^	.997^a^	.994^a^
Kyrgyzstan	0.9	.734^a^	.994^a^	.807^a^	.994^a^	.928^a^	.771^a^	−0.135230428	−.995^a^	.998^a^	.992^a^
Norway	0.9	.931^a^	.994^a^	.998^a^	.998^a^	−.970^a^	.983^a^	−0.116491378	−.900^a^	1.000^a^	.999^a^
Canada	0.9	.985^a^	.983^a^	.972^a^	.988^a^	−.814^a^	.990^a^	−0.384309068	−.956^a^	.993^a^	.991^a^
Mauritius	0.8	.989^a^	.994^a^	.986^a^	.997^a^	−.972^a^	.983^a^	−.982^a^	−.910^a^	.984^a^	.997^a^
Spain	0.8	.899^a^	.975^a^	.983^a^	.968^a^	.622^a^	.964^a^	.651^a^	−.838^a^	.989^a^	.963^a^
Switzerland	0.7	.967^a^	.987^a^	.951^a^	.997^a^	−.993^a^	.972^a^	−0.08072924	−.914^a^	.998^a^	.991^a^
Fiji	0.7	.983^a^	.994^a^	.993^a^	.994^a^	.461^b^	.998^a^	−.995^a^	−.525^a^	.992^a^	.994^a^
El Salvador	0.6	.973^a^	.936^a^	.938^a^	.934^a^	.731^a^	.963^a^	−.971^a^	−.985^a^	.984^a^	.940^a^
Thailand	0.6	.927^a^	.976^a^	.976^a^	.970^a^	−.965^a^	.840^a^	−.993^a^	−.996^a^	.991^a^	.993^a^
Sweden	0.6	.921^a^	.981^a^	.982^a^	.982^a^	−.873^a^	.963^a^	0.101445839	−.871^a^	.999^a^	.971^a^
Republic of Korea	0.6	.978^a^	.978^a^	.978^a^	.976^a^	0.037849082	.986^a^	−.887^a^	−.993^a^	.999^a^	.997^a^
Malta	0.5	.987^a^	.961^a^	.962^a^	.961^a^	0.261203029	.944^a^	−.904^a^	−.975^a^	.991^a^	.988^a^
United Kingdom	0.5	.951^a^	.998^a^	.989^a^	.999^a^	−.951^a^	.997^a^	.587^a^	−.949^a^	.993^a^	.990^a^
China	0.5	.847^a^	.887^a^	.807^a^	.971^a^	−.914^a^	.952^a^	−.778^a^	−.948^a^	.982^a^	.970^a^
Belgium	0.5	.911^a^	.968^a^	.963^a^	.970^a^	.851^a^	.960^a^	.766^a^	−.813^a^	.993^a^	.987^a^
France	0.5	.968^a^	.998^a^	.999^a^	.996^a^	.435^b^	.996^a^	.949^a^	−.890^a^	.999^a^	.980^a^
Austria	0.5	.962^a^	.975^a^	.973^a^	.969^a^	−0.082365879	.968^a^	−0.213468899	−.947^a^	.982^a^	.974^a^
Netherlands	0.5	.976^a^	.992^a^	.991^a^	.993^a^	−.996^a^	.954^a^	.797^a^	−.991^a^	.998^a^	.981^a^
Denmark	0.4	.952^a^	.982^aa^	.993^a^	.965^a^	−.928^a^	.976^a^	0.274251904	−.959^a^	.991^a^	.988^a^
Portugal	0.4	.897^a^	.998^a^	.995^a^	.998^a^	−0.330767899	.984^a^	−.856^a^	−.953^a^	.941^a^	.747^a^
Finland	0.4	.901^a^	.987^a^	.980^a^	.985^a^	−.866^a^	.994^a^	0.068426936	−.980^a^	.999^a^	.908^a^
Uruguay	0.3	.814^a^	.965^a^	.916^a^	.984^a^	−.702^a^	.988^a^	−.996^a^	−.975^a^	.989^a^	.974^a^
Italy	0.3	.617^a^	.944^a^	.936^a^	.948^a^	0.13089452	.919^a^	.855^a^	−.800^a^	.966^a^	0.052788676
Barbados	0.3	.943^a^	.993^a^	.985^a^	.990^a^	−.986^a^	.996^a^	.949^a^	−.615^a^	.994^a^	.998^a^
Kazakhstan	0.2	.594^a^	.719^a^	.793^a^	.579^a^	−.469^b^	.826^a^	.829^a^	−0.327880182	.978^a^	.762^a^
Czech Republic	0.2	.741^a^	.787^a^	.799^a^	.756^a^	−.717^a^	.728^a^	0.284642336	−.519^a^	.986^a^	.403^b^
Greece	0.1	.728^a^	.896^a^	.836^a^	.929^a^	−.909^a^	.825^a^	0.194590328	−.919^a^	.944^a^	.867^a^
Lesotho	0.1	−0.04098	−0.12795	−0.1696	− 0.09869	−0.187421016	.647^a^	0.324560341	−.707^a^	−0.15536	−0.125005334
Cuba	0.1	0.036673	0.356651	−.878^a^	.464^b^	.873^a^	.447^b^	−.878^a^	−.629^a^	.654^a^	.541^a^
Germany	0.1	0.084016	0.155109	0.089014	0.249967	−0.100457743	0.080313022	−.435^b^	−0.321908487	.749^a^	.536^a^
Slovakia	0.1	0.065469	0.294484	0.182123	0.2956	0.347932266	0.282911334	−.794^a^	−.405^b^	.701^a^	.403^b^
Poland	0.0	−.742^a^	−.700^a^	−.762^a^	−.645^a^	.676^a^	−.657^aa^	0.214183269	.555^a^	.543^a^	−.551^a^
Slovenia	−0.1	−.797^a^	−.912^a^	−.893^a^	−.923^a^	−.639^a^	−.912^a^	−.487^b^	.896^a^	−.754^a^	−.730^a^
Japan	−0.1	−.688^a^	−.657^a^	−.605^a^	−.693^a^	0.322439786	−.704^a^	−0.112441575	.691^a^	−.454^b^	.977^a^
Hungary	−0.2	−.937^a^	−.990^a^	−.966^a^	−.985^a^	.983^a^	−.974^a^	.778^aa^	.977^a^	.970^a^	.421^b^
Guyana	−0.3	−.740^a^	−.789^a^	−.721^a^	−.924^a^	−.753^a^	−.638^a^	.866^a^	.645^a^	−0.14194	−.907^a^
Romania	−0.3	−.863^a^	−.940^a^	−.846^a^	−.771^a^	.985^a^	−.898^a^	0.006284897	.959^a^	.977^a^	.857^a^
Russian Federation	−0.3	−.472^b^	−.497^b^	0.249803	−.966^a^	0.389953573	−0.189673631	0.139860835	.809^a^	.920^a^	−.935^a^
Trinidad and Tobago	−0.4	−.987^a^	0.244115	−.830^a^	−.980^a^	.973^a^	−.951^a^	.642^a^	.959^a^	−.982^a^	−.966^a^
Albania	−0.4	−.831^a^	−.860^a^	−.835^a^	−.863^a^	.713^a^	−.821^a^	.805^a^	.819^a^	.776^a^	−0.065109846
Croatia	−0.4	−0.38989	−.672^a^	−.652^a^	−.680^a^	−.650^a^	−.650^a^	.409^b^	.839^a^	.855^a^	.853^a^
Serbia	−0.5	− 0.34839	−.956^a^	−.849^a^	−.914^a^	−.855^a^	−.909^a^	−.580^a^	.956^a^	.914^a^	.858^a^
Armenia	−0.7	−.522^a^	−0.30719	−.470^b^	0.022436	−0.369420685	−.848^a^	.930^a^	.875^a^	.936^a^	.907^a^
Ukraine	−0.7	0.066367	.680^a^	.918^a^	−.855^a^	.636^a^	−0.39443992	0.345994432	.902^a^	.980^a^	.947^a^
Estonia	−0.9	−.768^a^	−.628^a^	−.781^a^	− 0.0765	−0.234462661	−.774^a^	0.254495629	.942^a^	.993^a^	.947^a^
Bulgaria	−0.9	−.566^a^	−.864^a^	−.702^a^	−.912^a^	.859^a^	−.770^a^	0.042146942	.863^a^	.991^a^	.925^a^
Republic of Moldova	−1.0	−0.11151	−.976^a^	.967^a^	−.977^a^	0.220245902	−.830^a^	.870^a^	.962^a^	.985^a^	−.997^a^
Lithuania	−1.2	−.826^a^	−.976^a^	−.971^a^	−.417^b^	−.570^a^	−.803^a^	0.294372658	.919^a^	.992^a^	.991^a^
Latvia	−1.3	−.649^a^	−.848^a^	−.905^a^	.882^a^	−.956^a^	−.878^a^	0.196002056	.935^a^	.997^a^	.985^a^

^a^. Correlation is significant at the 0.01 level (2-tailed)

^b^. Correlation is significant at the 0.05 level (2-tailed)

## Abbreviations

GDP: Gross Domestic Product
HAQ Index: Healthcare Access and Quality Index
IWI: Inclusive Wealth Index
LAC: Latin America and the Caribbean
LICs: low-income countries
LMICs: lower-middle income countries
SDGs: Sustainable Development Goals
SES: socioeconomic status
SSA: Sub-Saharan Africa
UMICs: upper-middle-income countries
UN: United Nations
VSL: value of statistical life
WHO: World Health Organization

## Footnotes

1 This assumption is popular due to the data availability of VSL. Specifically, we used the values of VSL only in 2000, as estimated in Viscusi and Joseph [26].

2 This assumption may fail to access the variations in health stock in developed countries because people over age 100 are significantly found in these countries. However, this assumption could simply compare national health stocks under the conditions of data availability of national life tables in 140 countries. Therefore, we leave this problem of requiring an elaborated estimation of health stock in developed countries to a future study.
